# CD40 ligand induced cytotoxicity in carcinoma cells is enhanced by inhibition of metalloproteinase cleavage and delivery via a conditionally-replicating adenovirus

**DOI:** 10.1186/1476-4598-9-52

**Published:** 2010-03-08

**Authors:** Taha Elmetwali, Peter F Searle, Iain McNeish, Lawrence S Young, Daniel H Palmer

**Affiliations:** 1 Cancer Research UK Institute for Cancer Studies, School of Cancer Sciences, University of Birmingham, B15 2TT, UK

## Abstract

**Background:**

CD40 and its ligand (CD40L) play a critical role in co-ordinating immune responses. CD40 is also expressed in lymphoid malignancies and a number of carcinomas. In carcinoma cells the physiological outcome of CD40 ligation depends on the level of receptor engagement with low levels promoting cell survival and high levels inducing cell death. The most profound induction of cell death in carcinoma cells is induced by membrane-bound rather than recombinant soluble CD40L, but like other TNF family ligands, it is cleaved from the membrane by matrix metalloproteinases.

**Results:**

We have generated a replication-deficient adenovirus expressing a mutant CD40L that is resistant to metalloproteinase cleavage such that ligand expression is retained at the cell membrane. Here we show that the mutated, cleavage-resistant form of CD40L is a more potent inducer of apoptosis than wild-type ligand in CD40-positive carcinoma cell lines. Since transgene expression via replication-deficient adenovirus vectors *in vivo *is low, we have also engineered a conditionally replicating E1A-CR2 deleted adenovirus to express mutant CD40L, resulting in significant amplification of ligand expression and consequent enhancement of its therapeutic effect.

**Conclusions:**

Combined with numerous studies demonstrating its immunotherapeutic potential, these data provide a strong rationale for the exploitation of the CD40-CD40L pathway for the treatment of solid tumours.

## Background

CD40, a member of the tumour necrosis factor receptor (TNFR) superfamily, and its ligand (CD40L/CD154) play a fundamental role in co-ordinating immune responses [1]. CD40 is expressed on normal B cells, monocytes and dendritic cells (DC) and interaction with its ligand promotes dendritic cell maturation, upregulation of co-stimulatory molecules and secretion of immunostimulatory cytokines. Thus, CD40 stimulation can effect the key elements required for generation of antigen-specific cytotoxic T-cell responses. On this basis, engagement of CD40 on DC to induce anti-tumour immune responses is a prolific area of research and both recombinant soluble CD40 ligand and CD40 agonist antibodies have entered clinical trials.

We, and others, have demonstrated that in addition to immune cells, CD40 is expressed in malignant haemopoietic cells and a number of carcinomas [2]. In carcinoma cells the level of CD40 engagement influences the physiological outcome with low levels of ligation promoting cell survival/proliferation and high levels inducing growth arrest/apoptosis [3-5]. The precise form of the CD40 stimulus affects these responses with the most profound effects in carcinoma cells being induced by membrane-bound (mCD40L) rather than recombinant soluble CD40L (rsCD40L) [6,7].

We have previously found that rsCD40L can stimulate survival signalling pathways (including PI-3-kinase and ERK/MAPK) and induces apoptosis in carcinoma cells only in the presence of either protein synthesis inhibition, cytotoxic drugs or inhibitors of the PI3K/mTOR and/or ERK pathways [8]. In contrast, membrane-bound CD40L delivered by co-culture of carcinoma cells with CD40L-expressing fibroblasts induces apoptosis without the requirement for any other agent [6,7]. Thus, as a potential anti-cancer therapy, membrane-bound CD40L appears to be more attractive than the recombinant soluble form. As a means of delivering membrane-bound CD40L in a form that may be clinically applicable, we have generated a replication-deficient recombinant adenovirus encoding human CD40L (RAdCD40L), which results in expression of ligand at the cell membrane. Further, based on our previous observation that Fas ligand mutated to resist cleavage from the cell membrane delivers a more potent apoptotic stimulus than wild-type FasL, we have generated a mutant CD40L that is resistant to cleavage by matrix metalloproteinases. The direct effect of wild-type and cleavage-resistant CD40L on cell survival was examined in CD40-positive carcinoma cell lines. Since transgene expression via replication-deficient adenovirus vectors *in vivo *is low, we have also engineered an E1A-deleted conditionally replicating adenovirus to express mutant CD40L with the aim of amplifying its expression and consequently its therapeutic effects.

## Methods

### Adenoviral construction and generation of CD40 ligand mutant

To generate a replication-deficient adenovirus expressing CD40L, human cDNA encoding wild-type CD40L was cloned in-frame under a CMV promoter into the pAdTrack-CMV vector. After confirming CD40L expression in HEK293 cells, this vector or the empty pAdTrack-CMV vector were homologously recombined with an E1-, E3- deleted adenoviral AdEasy vector as described by He et al (1998) to generate RAdCD40L or GFP control virus (RAdMock) [9]. Virus was packaged in the E1-expressing cell line, 911, and purified by caesium chloride banding. Virus titres were determined using the TCID_50 _method, based on the development of CPE in HEK293 cells using serial dilutions to estimate adenovirus stock titre.

To generate a CD40L mutant lacking the amino acid sequence (^110^SFEMQKG^116^) the Quick Change site-directed mutagenesis (Strategene Europe, Amsterdam, Netherlands) was utilized using forward: 5'GAGGAGACGAAGAAAGAAGATCAGAATCCTCAAATTGCGGC 3' and reverse: 5'GCCGCAATTTGAGGATTCTGATCTTCTTTCTTCGTCTCCTC 3' primers in a PCR reaction according to the manufacturer's instructions. Following sequencing of the deletion mutation, a RAd expressing the CD40L mutant (RAdncCD40L) was then generated as described above.

To generate replication-competent adenoviruses expressing either ncCD40L mutant or GFP, forward: 5'GTCCACTGTCGCCGCCACAAG 3' and reverse: 5'GCTGCGCCTTTGGCCTAATACC 3' primers were used to amplify the 634 bp DNA fragment from an Ad5E3 DNA construct (originally generated by cloning the 5665 bp of the HindIII fragment of Ad5 into the pUC19 plasmid) upstream of the 5' end of the AdE3 6.7 k fragment for replacing the 6.7 k and 19 k fragments of the AdE3 region. The amplified fragment contains a BsiWI site at the 5' position. To fuse the ncCD40L into the 634 bp amplified fragment, a ncCD40L forward primer: 5' GTATTAGGCCAAAGGCGCAGCTTCGAACCACCatgatcgaaacatacaaccaaacttc 3' containing 32 bp at the 5' end overlapping with the 3' end of the amplified 634 bp fragment and a reverse primer 5'CAGCAATTGGATCctgttcagagtttgagtaagccaaag 3' with an artificial MfeI site at the 3' region were used to amplify the ncCD40L from the pAdTrack-CMV-ncCD40L construct. The amplified ncCD40L was then fused to the 634 bp fragment of the AdE3 region utilizing the forward: 5'GTCCACTGTCGCCGCCACAAG 3' and the reverse: 5'CAGCAATTGGATCctgttcagagtttgagtaagccaaag 3' primers. The AdE3-ncCD40L fusion fragment was then cloned into the Ad5E3 DNA construct between BsiWI and MfeI restriction sites. The resultant construct was homologously recombined with an E1-deleted adenovirus in E. coli BJ5183 cells in order to replace the 6.7 k and 19 k fragments of the AdE3 region with the ncCD40L gene. To provide the E1-deleted AdncCD40L with an E1-encoding cDNA, a second homologous recombination was carried out in HEK 293 cells with a viral vector containing the AdE1 gene with a deletion mutation within the CR2 region (dl922-947). Following several rounds of adenoviral selection on human alveolar basal epithelial carcinoma A549 cells, which lack the E1 gene, replication competent adenoviruses expressing either ncCD40L mutant or GFP were extracted from single colony infection and amplified in A549 cells for adenoviral stock preparation.

For *in vitro *infections, cells were seeded in 10% FCS D-MEM complete media at 4,000 cells/well in a 96-well plate for cell viability assay, or 3 × 10^5 ^cells/30 mm dish for western blotting analysis. Cells were infected in 10% FCS D-MEM for 2 hours at 37°C with the appropriate MOI of adenovirus. Culture medium was then replaced by fresh medium and cells incubated at 37°C until assessed.

### RNA extraction and RT-PCR analysis

RNA was extracted using the RNAzol reagent (Biogenesis Ltd, Poole UK) and cDNA was synthesised using RETROscript^® ^RNase reverse transcription kit (Ambion Europe) according to the manufacturer's instructions. RT-PCR was performed using PLATINUM™ Taq DNA polymerase (Invitrogen) utilising the human CD40-specific forward: 5'AGTGACTGCACAGAGTTCACT 3' and reverse 5'CAAGAGGATGGCAAACAGGAT 3' primers. To confirm the CR2 mutation deletion (dl922-947), forward primers, 5' CTGCCACGAGGCTGGC 3' and 5' CCACACACGCAATCACAGG 3' spanning either a DNA sequence within the CR2-deletion (dl922-947) or downstream of the deletion respectively were used in RT-PCR utilizing the general reverse primer 5' CCACACACGCAATCACAGG 3' to discriminate adenovirus carrying wild type E1A CR2 from that carrying the E1A CR2 deletion mutant. For RT-PCR, the amount of cDNA template used was adjusted on the basis of amplification with primers specific for human GAPDH: forward 5' CCTCCAAAATCAAGTGGGGCG 3' and reverse 5'ACCACCAGGTGCTCAGTGTAG 3'.

### Pharmacological inhibitors

Cells were seeded in 10% FCS D-MEM complete media at 4,000 cells/well in a 96-well plate infected with the appropriate MOI of either RAdMock or RAdCD40L or left uninfected as a control. Twenty-four hours post-infection cells were treated with 30 μM of the metalloproteinase inhibitor, MMPI II (Calbiochem) or left untreated for a further 24 hours. Cell viability was then determined by WST-1 assay.

### WST-1 cell viability assay

Cells were plated in 96-well plates at 4000 cell/100 μl/well and incubated at 37°C. Cell viability was assessed by adding WST-1 reagent (Roche) to the culture medium at 1:10 dilution. Cells were incubated at 37°C and the optical density was measured by microplate ELISA reader at λ_450 _every 2 hours to a maximum of 6 hours. The amount of the formazan formed directly correlates to the number of metabolically active cells.

### Analysis of cell surface CD40L expression

For CD40L expression, cells were infected with RAdCD40L or RAdncCD40L at 100 MOI for 24 hours. Cells were washed and incubated on ice for 45 minutes with mouse anti-CD40L-APC conjugate antibody or mouse isotype-APC antibody conjugate or left without treatment. Cells were then washed, fixed in 1% PFA and analysed by flow cytometry.

### Annexin V staining of apoptotic cells

Following the relevant treatment, cells were harvested and stained with Annexin V-APC according to the manufacturer's instruction (BD Biosciences). The percentage of Annexin V-APC positive cells was determined within 1 × 10^4 ^cells of the population by flow cytometry.

### sCD40L enzyme-linked immunosorbent assay (ELISA)

EJ cells were plated in 96-well plates at 4000 cell/100 μl/well and incubated at 37°C in 10% FCS D-MEM medium. Culture media were collected from RAdCD40L, RAdncCD40L, RAdMock, uninfected control and cell-free medium control 24 and 72 hours post-infection and analysed for human sCD40L by ELISA assay (Biosource International, USA) according to the manufacturer's instructions.

### Western blot analysis and antibodies

Anti-CD40L antibody was from Santa Cruz Biotechnology. Monoclonal mouse anti- β-actin and GFP antibodies were from Sigma and Sterillin, UK respectively. For immunoblotting, 10-30 μg protein was separated by SDS-PAGE and transferred onto nitrocellulose membranes and blocked with 5% non-fat milk. Membranes were incubated overnight at 4°C with primary antibody and after washing, incubated for 1 hour at room temperature with the appropriate secondary antibody. Membranes were washed followed by enhanced chemiluminescence (Amersham Biosciences).

## Results

### Adenovirus-delivered CD40L directly induces apoptosis in CD40 positive carcinoma cells

**Figure 1:**
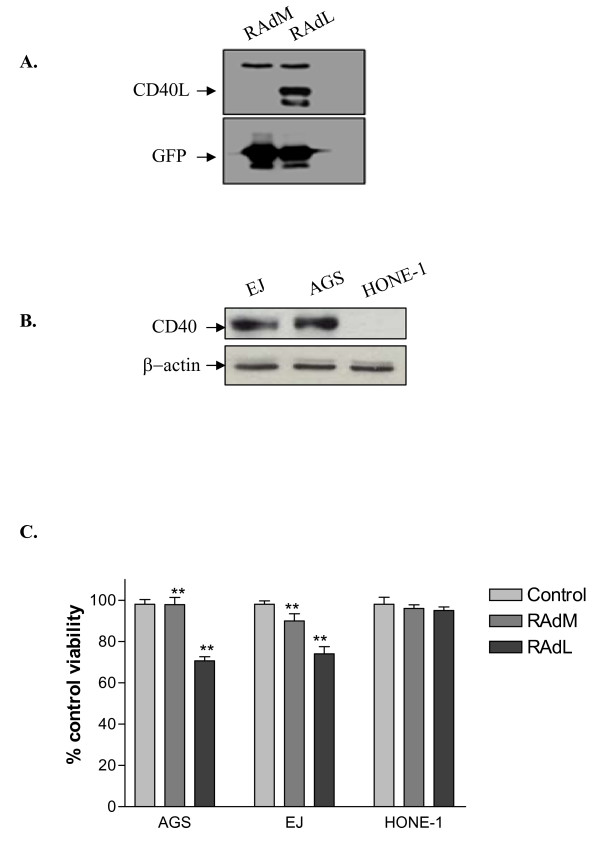
**Adenovirus-delivered CD40L directly induces growth inhibition in CD40 positive carcinoma cells**. (A) Analysis of CD40L and GFP expression from RAdCD40L and RAdMock viruses. HEK 293 cells were infected with RAdMock or RAdCD40L. Two days later cell lysates were probed with anti-CD40L and anti-GFP specific antibodies. (B) Analysis of the expression of CD40 receptor. 30 μg of total protein lysate from the carcinoma cell lines EJ, AGS and HONE-1 were examined for CD40 receptor expression by Western blotting using a specific anti-CD40 antibody. β-actin expression was determined as a loading control. (C) Effect of RAdCD40L on cell viability. The cancer cell lines AGS, EJ and HONE-1 were infected with 25 or 100 MOI of either RAdMock (RAdM) or RAdCD40L (RAdL) or left without infection. Forty-eight hours later cell viability was assessed by WST-1 assay. Results are the mean of triplet samples +/- SD. Two-tailed t-test RAdMock vs. RAdL: AGS, p = 0.0011; EJ, p = 0.0034; HONE-1, p = 0.4818.

We first constructed a replication-deficient adenovirus to express human wild-type CD40 ligand, designated RAdCD40L, using the AdEasy system (Quantum Biotechnologies) and methods as published by Chuan He and co-workers [9]. Expression of CD40L from this vector was confirmed by western blotting for CD40L protein in lysates of infected cells (Figure 1A).

The effect of RAdCD40L on the viability of a panel of epithelial cell lines was then examined. The bladder carcinoma line, EJ, and the gastric carcinoma line, AGS, are CD40 receptor-positive, whilst the CD40-negative nasopharyngeal carcinoma line, HONE-1, was used as a negative control (Figure 1B). Each cell line was infected with either RAdMock or RAdCD40L or left uninfected as a negative control and 48 hours later cell viability was assessed by WST-1 assay. RAdCD40L infection induced a modest reduction in viability of the CD40-positive EJ and AGS but, as expected, had no effect on the CD40-negative HONE-1 cells (RAdMock vs. RAdCD40L: AGS, p = 0.0011; EJ, p = 0.0034; HONE-1, p = 04818) (Figure 1C).

### Inhibition of CD40L cleavage from the cell membrane enhances its cytotoxicity

Ligands of the TNF family are biologically active as self-assembling, non-covalently bound trimers that are naturally expressed on the cell surface as membrane-bound structures but are proteolytically cleaved into soluble forms by members of the ADAM metalloproteinase (MP) family, a process which may serve a negative regulatory function in the signalling induced through receptor ligation [10]. Indeed, we have previously found that a RAd expressing a mutated FasL lacking the MP cleavage site is a much more potent inducer of carcinoma cell death than wild type FasL [11].

Since CD40L is also subject to proteolytic cleavage we hypothesised that inhibition of CD40L cleavage may enhance its anti-tumour effects [12]. In this study we have sought to identify the CD40L MP cleavage site with a view to generating mutated CD40L that is resistant to cleavage from the cell membrane.

**Figure 2:**
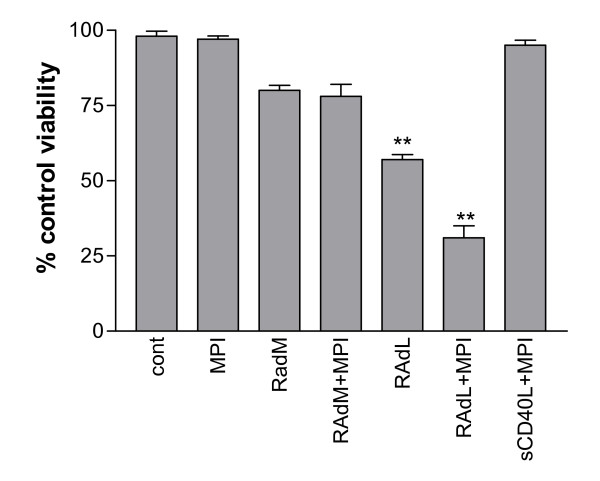
**The effect of matrix metalloproteinase inhibitors (MPI) on RAdCD40L-induced cell death**. EJ cells were infected with 100 MOI of either RAdMock (RAdM) or RAdCD40L (RAdL) or left without infection, or cultured with 1 μg/ml soluble CD40L. Twenty-four hours later cells were treated with 30 μM of MPI II or left without treatment for a further 24 hours. Cell viability was assessed by WST-1 assay. Results are the mean of triplet samples +/- SD. Two-tailed t-test RAdL vs. RAdL+MPI: p = 0.0041.

In order to first confirm that inhibition of MP cleavage of CD40L does, indeed, enhance its cytotoxicity, EJ cells were infected with RAdCD40L and 24 hours later were treated with MPI II, a potent, reversible, broad-range inhibitor of a number of MPs including MP-1, MP-3, MP-7, and MP-9. Treatment of RAdCD40L-infected cells with MPI II resulted in a significant incremental increase in cell death compared to RAdCD40L-infected cells in the absence of MPI (p = 0.0041). In uninfected cells MP inhibition alone did not affect cell viability. Moreover, and as expected, MPI II had no effect on the viability of cells treated with rsCD40L (Figure 2). These results strongly suggest that CD40L is a more potent inducer of carcinoma cell death when its expression is retained at the cell membrane. On this basis, we next sought to identify the CD40L cleavage site that is targeted by MP enzymes.

### Identification and mutation of the CD40L cleavage site

**Figure 3:**
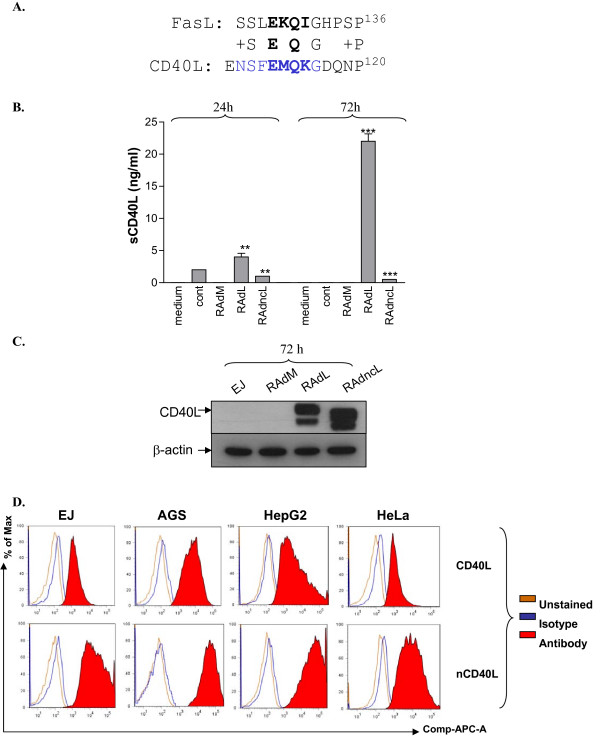
**Identification and mutation of the CD40L cleavage site leads to retention of ligand at the cell membrane**. (A) Amino acid alignment of FasL and CD40L. Alignment identified a region in the extracellular domain of CD40L (EMQK) with significant similarity to the cleavage site of FasL (EKQI). CD40L mutant (ncCD40L) is not cleaved into soluble CD40L. EJ cells were infected with 100 MOI of either RAdMock (RAdM) or RAdCD40L (RAdL) or RAdncCD40L (RAdncL) or left without infection. (B) Samples were collected from the culture media 24 and 72 hours post-infection for sCD40L ELISA assay. Results represent a mean of triplet samples +/- SD. Two-tailed t-test RAdL vs. RAdncL: 24 h, p = 0.0067; 72 h, p < 0.0001. (C) CD40L expression was examined by western blot 72 hours after infection. β-actin was used as a loading control. (D) Adenovirus-delivered CD40L is expressed on the cell membrane. EJ cells were infected with 100 MOI of either RAdCD40L (wild-type) or RAdncCD40L (mutated cleavage resistant) for 28 hours. Harvested cells were incubated with mouse anti-CD40L-APC conjugated antibody or isotype control and then analysed by flow cytometry.

Previous studies have shown that deletion of the Fas ligand region that includes amino acid 128-131 retains its receptor binding capacity but blocks release of soluble FasL from the cell membrane [11]. To identify the putative MP cleavage site within CD40L, we performed amino acid sequence alignment between the published FasL cleavage site and CD40L, identifying a region in the extracellular domain of CD40L with significant homology with the known cleavage site of FasL (^128^EKQI^131^) (Figure 3A). In addition to this region of homology, more supportive information was obtained from published studies of bacterial expression and purification of the CD40L extracellular domain (44-261 amino acids), which was consistently associated with two protein fragments running at molecular weights of 18- and 14-kDa on SDS-PAGE electrophoresis. Further purification and amino acid sequence analysis showed that the 18-kD fragment was active in the context of CD40 biology and that this sequence starts from the amino acid ^108^ENSFEMQKG^116^, which contains the same sequence identified by alignment with the FasL cleavage site [13]. These observations suggest this to be the most likely CD40L cleavage site. On this basis, we next generated a mutant CD40L with an amino acid deletion mutation encompassing the ^110^SFEMQKG^116 ^sequence and engineered this into a RAd vector.

To generate a clone of pAdTrack-CMV-CD154 (CD40L) with the amino acid sequence ^110^SFEMQKG^116 ^deletion mutation we utilised the quickchange site-directed mutagenesis (SDM) reagent as described in Materials and Methods utilising forward and reverse primers of CD40Lde110-116F and CD40Lde110-116R spanning the targeted deletion site. Sequence analysis confirmed successful generation of the deletion mutation and the mutant sequence was cloned into a replication-deficient adenovirus vector, designated RAdncCD40L.

To confirm that the deletion mutant was resistant to MP cleavage, EJ cells were infected with RAdCD40L or RAdncCD40L and supernatant was harvested and analysed for presence of soluble ligand using a sCD40L ELISA assay (Figure 3B). CD40L protein expression was also assessed by western blotting of cell lysates from the same samples (Figure 3C). A significant quantity of sCD40L was detected in the supernatant of RAdCD40L infected cells with the amount increasing over time. However, no soluble ligand was detected in the supernatant of RAdncCD40L infected cells, confirming that deletion of the ^110^SFEMQKG^116 ^sequence blocked cleavage of CD40L from the cell membrane despite high levels of ligand expression on western blotting (RAdCD40L vs. RAdncCD40L at 72 hours, p < 0.0001). The level of ncCD40L protein was higher than that of wild-type ligand assessed by western blot analysis suggesting that a significant amount of CD40L expressed from the RAdCD40L was released into the culture medium. Furthermore, retention of CD40L at the cell membrane was confirmed by flow cytometry. A panel of cell lines infected with wild-type CD40L demonstrated ligand expression at the membrane. However, infection with the equivalent MOI of non-cleavable CD40L resulted in significantly greater membranous ligand expression (Figure 3D).

**Figure 4:**
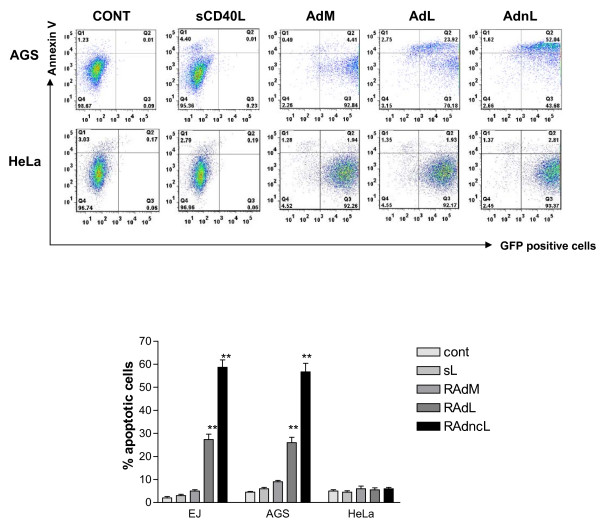
**Membrane-bound expression of CD40L induces apoptosis in CD40-positive carcinomas**. EJ, AGS and HeLa cells were infected with 100 MOI RAdMock, RAdCD40L or RAdncCD40L, or treated with soluble CD40L (1 μg/ml) or left without treatment for 48 hours. Apoptotic cells were assessed by staining with Annexin V-APC and analysis by flow cytometry. Results are a mean of triplet samples ± SD. Two-tailed t-test RAdL vs. RAdncL: EJ, p = 0.0014; AJS, p = 0.0022; HeLa, p = 0.6560.

We next sought to investigate the ability of membrane-bound CD40L to induce apoptosis in CD40-positive carcinomas. Unlike soluble CD40L, RAd-delivered CD40L is able to directly induce apoptosis in CD40-positive carcinoma cells (EJ, AGS) as assessed by Annexin V staining. Furthermore, significantly higher levels of apoptosis are induced by cleavage-resistant ligand (RAdncCD40L vs. RAdCD40L: EJ, p = 0.0014; AGS, p = 0.0022). The requirement for CD40-CD40L interaction for apoptosis was confirmed by an absence of effect in CD40-negative HeLa cells (Figure 4; p = 0.6560).

Thus RAdncCD40L produces CD40 ligand expressed on the membrane of infected cells, fails to generate soluble ligand and demonstrates significantly enhanced apoptosis induction compared to RAdCD40L without the requirement for protein synthesis inhibition.

### Delivery of membrane-bound CD40L via a conditionally replicating adenovirus

Here we have demonstrated the ability of a mutant non-cleavable CD40L delivered via a replication-deficient adenovirus vector to directly induce cell death in CD40 expressing carcinoma cells. However, it is recognised that such vectors generally result in low-level transgene expression *in vivo*. We hypothesised that the generation of a replication-competent adenovirus expressing the ncCD40L would have significantly greater cytotoxic effects through amplified expression of the membrane-bound ligand and dissemination within the tumour environment to enable the paracrine stimulation of CD40, as well as through the effects of viral oncolysis.

For wild-type adenovirus to replicate normally the viral E1A early protein, through its CR2 domain, is required to bind to and inactivate the retinoblastoma protein (pRb) in the infected host cell in order to subvert anti-viral growth arrest responses. Thus, deletion of this domain abrogates viral replication in normal cells with an intact pRb response. However, in most human cancers the pRb pathway is perturbed. As a result of this, replication of an adenovirus with a CR2 dl922-947 deletion mutation in the E1A gene is restricted to tumour cells and virus replication results in tumour cell lysis, propagating infective virus throughout the tumour [14].

**Figure 5:**
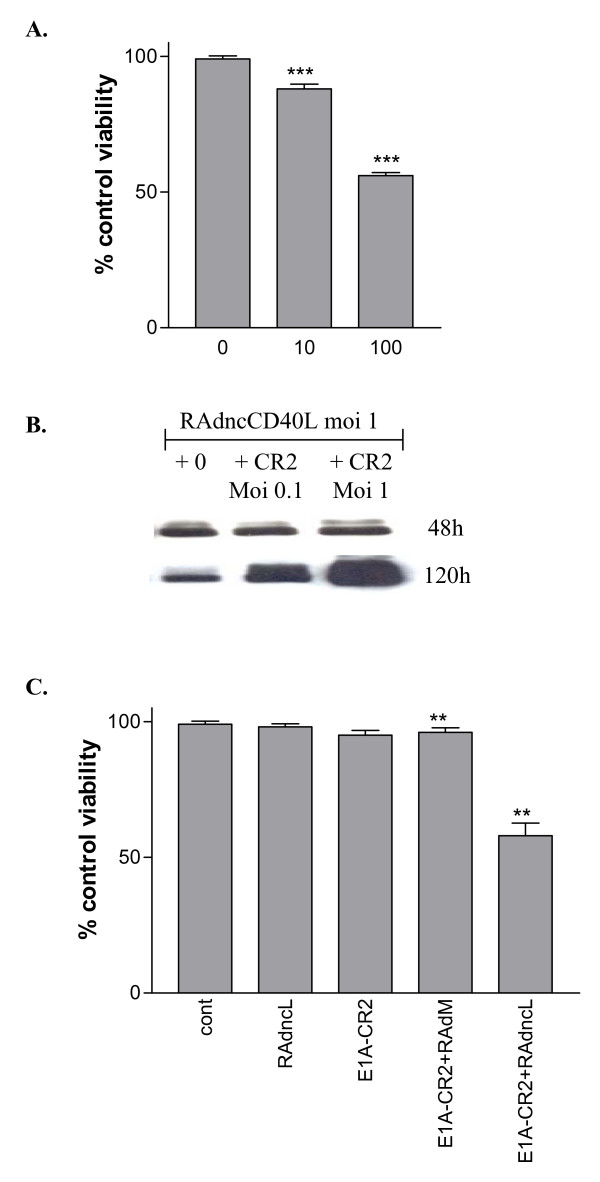
**RAd-delivered CD40L acts in synergy with conditionally replicating adenovirus**. (A) RAdncCD40L cytotoxicity is dose-dependent. EJ cells were infected with 0, 10 or 100 MOI RAdncCD40L and 48 hours later cell viability was assessed by WST-1 assay. Results are the mean of triplet samples +/-SD. Two-tailed t-test 10 MOI vs. 100 MOI: p = 0.0001. Co-infection with RAdncCD40L and E1AΔCR2 enhances ncCD40L expression and cytotoxicity. (B) EJ cells were infected with 1 MOI RAdncCD40L and co-infected with 0, 0.1 or 1 MOI E1AΔCR2. Cell lysates were harvested at 48 and 120 hours and 20 μg total protein was blotted for ncCD40L. (C) EJ cells were plated in 96-well plates and infected with 1 MOI of either RAdncCD40L or RAdMock or left uninfected; simultaneously cells were infected with 0.1 MOI E1A ΔCR2 and 120 hours later cell viability was assessed by WST-1 assay. Results are the mean of triplet samples +/-SD. Two-tailed t-test E1A-CR2+RAdM vs. E1A-CR2+RAdncL: p = 0.0015.

In order to establish the rationale for its incorporation into a replicating adenovirus, we first demonstrated that the degree of cell death induced by ncCD40L is dose-dependent indicating that amplification of its expression will increase cell death (RAdncCD40L 10 MOI vs. 100 MOI: p = 0.0001) (Figure 5A). This was further corroborated by co-infecting EJ cells with RAdncCD0L and an E1A CR2-deleted adenovirus (E1A-ΔCR2). The dose of each virus individually was insufficient to induce cell death. However, the combination resulted in increased expression of CD40L presumably through viral proteins expressed by the E1A-ΔCR2 supporting replication of the RAd, and which resulted in cell death (E1A-CR2+RAdMock vs. E1A-CR2+RAdncCD40L: p = 0.0015) (Figure 5B and 5C).

**Figure 6:**
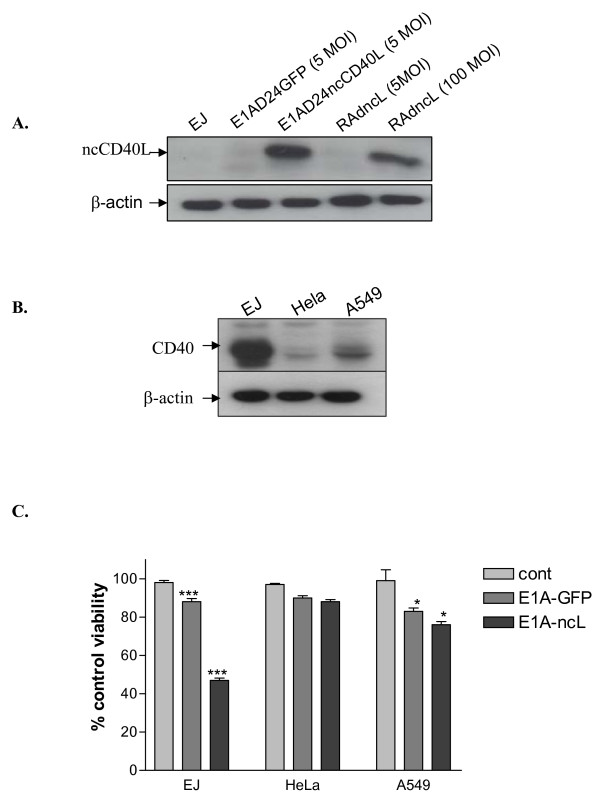
**Delivery of membrane-bound CD40L via a conditionally replicating adenovirus**. (A) Expression of ncCD40L by E1AD24ncCD40L. A549 cells were infected with 5 MOI E1AD24ncCD40L or E1AD24GFP or left uninfected for 48 hours. In parallel, cell were infected with RAdncCD40L (5 or 100 MOI). 10 μg of total protein lysates were blotted for ncCD40L. (B) Analysis of CD40 expression. Total protein lysates from EJ, Hela and A549 cells were harvested and CD40 expression was examined by western blot. β-actin was used as a loading control. (C) Effect of E1AD24ncCD40L on cell viability. EJ, Hela and A549 cells were infected with 5 MOI of either E1AD24GFP (E1AGFP) or E1AD24ncCD40L (E1AncL) or left without infection for 36 hours. Cell viability was then assessed by WST-1 assay. Results are the mean of triplet samples +/- SD. Two-tailed t-test E1A-GFP vs. E1A-ncL: EJ, p < 0.0001; HeLa, p = 0.2879; A549, p = 0.0460.

On this basis, we generated a CR2 dl922-947 deleted adenovirus expressing the ncCD40L, designated E1AD24ncCD40L, by replacing the 6.7 k and the 19 k fragments within the E3 region with the ncCD40L gene, driving its expression from the endogenous E3 promoter. Expression of ncCD40L from E1AD24ncCD40L was verified by immunoblotting lysates of infected EJ cells. Further, the level of ligand expression was compared with that achieved by the replication-deficient vector. The replicating vector resulted in significantly greater expression of CD40L, with an MOI of 5 exceeding the level achieved by even 100 MOI RAdncCD40L (Figure 6A).

We next examined the effect of E1AD24ncCD40L on the viability of CD40-positive carcinomas. Firstly, the CD40 status of bladder carcinoma cells, EJ, Hela cells and human alveolar basal epithelial carcinoma cells, A549, was determined. The level of CD40 expression in A549 cells was very low compared to EJ cells and, as expected, Hela cells did not express CD40 at all (Figure 6B). These cell lines were then infected with either E1AD24GFP or E1AD24ncCD40L and cell viability was assessed. E1AD24ncCD40L infection resulted in significant cell death in EJ cells and to a lesser extent in A549 cells in a time-dependant manner consistent with increasing ligand expression over time, and in proportion with the degree of CD40 receptor expression in these cell types (E1AD24GFP vs. E1AD24ncCD40L: EJ, p < 0.0001; A549, p = 0.0460). No significant cell death was observed in the CD40-negative Hela cells (p = 0.2879), the time points studied being too early for viral oncolysis, further confirming that the cell death in EJ is likely to be due to the robust expression of ncCD40L achieved by this vector (Figure 6C).

## Discussion

We have generated an E1-, E3- deleted replication deficient adenovirus expressing CD40L and shown that CD40-positive carcinoma cell lines are sensitive to CD40L-induced cell death. Cell death was further enhanced by inhibition of matrix metalloproteinases indicating that membrane-bound ligand is likely to be more potent in inducing carcinoma cell death. In common with other TNF ligands, CD40L is exposed to cleavage by the action of MPs, liberating soluble CD40L [12]. Previous studies have generated a mutated FasL lacking the metalloproteinase cleavage site, which is a more potent inducer of carcinoma cell death than wild type FasL [11]. To generate a non-cleavable CD40L mutant we identified the CD40L cleavage site by alignment of the published amino acid sequence of the FasL cleavage site and the CD40L sequence. This identified the ^108^ENSFEMQKG^116 ^region in the extracellular domain of CD40L, which exhibited significant homology with the cleavage site of FasL (^128^EKQI^131^). Along with other corroborative evidence, this encouraged us to generate a mutant CD40L with this sequence deleted by site directed mutagenesis. A RAd expressing this mutant was then constructed (RAdncCD40L) and its resistance to MP cleavage was confirmed. As expected this virus failed to generate soluble ligand, resukted in a higher level of membrane-bound ligand expression, and induced greater cell death than wild-type ligand in CD40-positive carcinoma cells.

Soluble CD40L is known to induce transient signalling including both pro-survival and pro-apoptotic signals, and induction of cell death in this context requires inhibition of *de novo *protein synthesis to inhibit survival signals and promote apoptosis [6,15-17]. Membrane-bound CD40L, on the other hand, through sustained activation of TRAF3-dependent JNK signalling and suppression of TRAF6-dependent survival signals, is able to directly induce cell death without the requirement for protein synthesis inhibition [[7], and Elmetwali et al, J Immunol, 2010. In Press]. Thus, the greater cytotoxicity of ncCD40L is likely to be due to retention of ligand at the cell membrane inducing more sustained pro-apoptotic signals than wild-type CD40L, the latter being susceptible to cleavage into soluble CD40L, a process that may serve a regulatory role in physiological TNF family ligand signalling.

In order to further enhance the anti-cancer potency of ncCD40L, we predicted that a replication-competent adenovirus expressing ncCD40L would induce greater cytotoxicity than a replication-deficient vector by increasing expression and dissemination of transgene as well as through the contribution of viral oncolysis.

For human adenovirus replication, the conserved region 2 (CR2) domain within E1A is required to mediate pRb binding and inactivation [18,19]. Thus, a deletion mutation, dl922-947, within CR2 permits virus replication in a broad range of cancer cells with abnormalities in cell-cycle checkpoints [17]. We have generated a dl922-947 mutant adenovirus and further modified it to express ncCD40L by replacing the 6.7 k and 19 k fragments of the E3 region. The E3 transcription unit is an ideal site for transgene insertion since it is non-essential for viral replication *in vitro *[20-22]. The 6.7 k and the 19 k fragments were chosen as a site for insertion of the ncCD40L gene because they are adjacent to one another in the viral genome, are encoded by the same E3 mRNA, and have overlapping open reading frames [23]. The E3 6.7 k protein functions in concert with adenoviral receptor internalization and degradation complex (RID) to reduce TRAIL receptor-1 and -2 cell surface expression [24]. Since CD40L cytotoxicity may, at least in part, be mediated through up-regulation of TNF family ligands including TRAIL, the deletion of E3 6.7 K may be advantageous to CD40L-mediated effects through retention of TRAIL receptor expression (17). The 19 k protein is known to bind MHC class I molecules in the endoplasmic reticulum (ER), inhibiting their transport to the infected cell surface [25]. Therefore, wild-type virus is able to evade detection and eradication by Ad-specific cytotoxic T lymphocytes (CTL). Thus, the loss of the 19 k protein should ensure that normal cells infected with the virus are 'visible' to host CTLs and efficiently eradicated. Incorporation of ncCD40L into this vector resulted in dramatically increased ligand expression compared to the replication-deficient counterpart and this translated into significantly greater induction of cell death in a CD40-dependent manner.

Previous studies have demonstrated that CD40 ligation on carcinoma cells induces up-regulation of antigen processing and presentation with consequent enhanced recognition of these cells by specific cytotoxic T lymphocytes and induces strong systemic anti-tumour cytotoxic T cell activity in tumour bearing mice. Further, co-delivery of immature dendritic cells with RAdCD40L induces strong anti-tumour immunity, while treatment with immature DCs alone has no effect in a number of tumour models [26-29]. Our demonstration of the ability of adenovirus-delivered CD40L to suppress proliferation and induce apoptosis in carcinoma cells provides further rationale for exploiting the CD40-CD40L pathway for the treatment of solid tumours and suggests an advantage over other immunostimulatory cancer therapy approaches, particularly through generation of a membrane-bound mutant and through enhanced delivery via a conditionally-replicating vector. Taken together, the CD40 pathway provides an opportunity to harness different anti-cancer approaches into one therapy and offers an attractive option for clinical trials. Our findings demonstrate clear advantages of our mutant CD40L compared to rsCD40L in terms of directly inducing death in infected cancer cells.

## Competing interests

The authors declare that they have no competing interests.

## Authors' contributions

TE contributed to the conception and design of the study, conduct of experiments, interpretation of data and manuscript preparation.

PFS and IM contributed to the concept, design and construction of conditionally replicating adenoviruses.

LSY contributed to the conception and design of the study, interpretation of the data and manuscript preparation.

DHP contributed to the conception and design of the study, conduct of experiments, analysis and interpretation of data and drafted the manuscript.

All authors have read and approved the final manuscript.

## Disclosures

No potential conflicts of interest to declare.
